# Whole-Genome SNP Characterisation Provides Insight for Sustainable Use of Local South African Livestock Populations

**DOI:** 10.3389/fgene.2021.714194

**Published:** 2021-10-28

**Authors:** Esté van Marle-Köster, Simon Frederick Lashmar, Anel Retief, Carina Visser

**Affiliations:** Department of Animal Science, University of Pretoria, Pretoria, South Africa

**Keywords:** cattle, genetic diversity, inbreeding, indigenous, sheep

## Abstract

Local cattle and sheep populations are important for animal production and food security in South Africa. These genetic resources are well adapted to the diverse climatic conditions and hold potential to be utilized in production systems subjected to climate change. The local beef breeds are well integrated into commercial livestock production systems with access to performance recording and genetic evaluations, while local sheep breeds are mainly utilised in smallholder and communal systems. The GeneSeek^®^ Genomic Profiler™ Bovine 150 K SNP genotyping array was used to evaluate the diversity and inbreeding status of four indigenous (Boran, Drakensberger, Nguni, Tuli), two composite (Bonsmara and Beefmaster) and two exotic (SA Hereford and Charolais) beef breeds. The Illumina^®^ Ovine 50 K SNP BeadChip was used to investigate five indigenous (Black Head Persian, Damara, Fat tail, Namaqua Afrikaner, Pedi) and three commercial (Dorper, Dohne Merino and SA Merino) populations. Although ascertainment bias was indicated by the low MAF (the autosome-wide proportion of SNPs with MAF< 0.05 ranged from 6.18 to 9.97% for cattle, and 7.59–13.81% for sheep), moderate genomic diversity was observed (mean H_o_ ranged from 0.296 to 0.403 for cattle, and 0.327 to 0.367 for sheep). Slightly higher levels of ROH-based inbreeding were calculated for cattle (F_ROH_ range: 0.018–0.104), than for sheep populations (F_ROH_ range: 0.002–0.031). The abundance of short ROH fragments (mean proportion of <4 Mb fragments: 0.405 for cattle, and 0.794 for sheep) indicated ancient inbreeding in both species. The eight cattle populations were categorized into indicine, taurine or Sanga subspecies based on principal component, model-based clustering and phylogenetic analyses, with high levels of admixture observed within the Drakensberger, Nguni and Tuli breeds. Within the sheep populations, a clear distinction could be seen between the dual-purpose breeds, the meat breed and the indigenous breeds. Despite directional selection practiced in the cattle breeds, genomic diversity was moderate with low inbreeding. The non-commercialized, indigenous sheep populations are more vulnerable with small effective populations. These results emphasise the value of genomic information for effective management to exploit the potential contribution of local genetic cattle and sheep resources in a changing environment.

## Introduction

For more than a century, indigenous and local cattle and sheep populations have contributed to the livelihood of South Africans through commercial, communal and smallholder production systems. Livestock production is under increased pressure to implement sustainable practises, which require careful consideration of production systems, climate change, as well as animal and human interactions (Stringer et al., 2020). Climate change is predicted to have a significant effect on agricultural practices and the livelihoods of the entire human population. The effects of increased temperature and more frequent droughts should favour selection of more adapted livestock resources. In Africa, cattle, sheep and goats are primary species for food production and security and include a number of adapted local breeds such as the N’Dama (Mwai et al., 2015) and fat-tailed sheep (Wilson, 2011). Local genetic resources are often praised for their unique adaptive traits, but at the same time neglected in national strategies due to perceived low performance (Mapiye et al., 2019).

In South Africa (SA), local Sanga (*Bos taurus africanus*) cattle populations, which include the Nguni and Tuli are found in commercial, smallholder and communal production systems (Van Marle-Köster and Visser, 2018). The Drakensberger, one of the oldest cattle breeds in SA with an official breed Society founded in 1947, is regarded as a SA indigenous breed (www.drakensbergers.co.za). Breed societies for Nguni and Tuli were founded in 1986 and 1970 respectively (Bester et al., 2003; Tuli Cattle, 2021), while the Boran (Zebu) was introduced to SA from Kenya and Uganda during the late 1990s and has become a popular choice for crossbreeding in the sub-tropical regions of SA. These breeds, similar to the locally developed SA Bonsmara and exotic breeds (SA Beefmaster, SA Hereford, SA Charolais), are well established in the seed stock and commercial livestock sectors with access to national animal recording, genetic evaluation and markets.

Small ruminants in developing countries constitute 56% of the all the domesticated ruminants in the world and are mostly located in semi-arid and arid areas (Akinmoladun et al., 2019). The continued danger of an uncertain water supply in these areas has already enforced a move from cattle to small ruminant production (Akinmoladun et al., 2019). This change was mostly motivated by these breed’s ability to produce a range of products (meat, milk and fibres), their short generation intervals and lower maintenance requirements, as well as their superior ability to cope with droughts and high temperatures (Benhin, 2006; Rust and Rust, 2013).

In contrast to local SA beef cattle breeds found in all production systems, local sheep breeds tend to be restricted to smallholder farming, while industrial breeds (mainly Merino types and Dorpers) are reared on commercial farms and used in intensive systems (Molotsi et al., 2017). Smallholder and communal farmers mainly keep non-descript indigenous populations (Dzomba et al., 2020). These populations contribute significantly towards food security and the socio-economic livelihoods of rural communities, as they typically have superior adaptive characteristics and relatively low maintenance requirements and feed intake (Kunene et al., 2011; Kunene-Ngubane, 2015; Molotsi et al., 2020).

The indigenous breeds include the Zulu-type sheep, of which the Pedi is one of the recognised ecotypes. Pedi sheep are well-known for their natural tolerance of external parasites and other diseases (Kunene et al., 2009). The Pedi, Namaqua Afrikaner and East African Damara are all considered fat-tailed breeds. The fat rumped Blackhead Persian was introduced from Somalia as a hair breed, but is well-equipped to tolerate the harsh South African environment and is mainly kept as a mutton breed (Dzomba et al., 2020). Both the Damara and Blackhead Persian are regarded as indigenous, transboundary breeds (Molotsi et al., 2020).

Local sheep types tend to be under-utilised in smallholder communities and neglected in the commercial sector. Little or no genetic improvement has been made over the past decades, and there are still very limited resources available to assist communities to farm productively with the indigenous sheep populations. The effective population sizes are relatively small and the census numbers of the breeds are generally declining (Kunene et al., 2009).

Genomic information can assist in elucidating the current genetic composition of the indigenous populations as well as the ancestry of both cattle and sheep populations. Microsatellite markers have been used to demonstrate some differences among the SA Nguni ecotypes (Sanarana et al., 2016; Madilindi et al., 2020) and confirmed genetic diversity in Tuli and Drakensberger populations (Van der Westhuizen et al., 2020). A series of SNP-based methodologies, including those related to genetic diversity (Makina et al., 2014; Makina et al., 2016), have also been applied to Afrikaner, Drakensberger and Nguni breeds, however, including ≤50 samples genotyped for sparser marker densities. Genetic diversity has also been studied in local sheep populations (Soma et al., 2012; Nedambale et al., 2020). SA beef breeds in general have been included in a 3-year national beef genomic program (BGP) (Walsh and Spazzoli, 2018) for building training populations for genomic selection. Therefore, genotypic information is more readily available for the local cattle breeds compared to small stock for which no national genotyping strategy exists.

In the small stock industry, the combination of small population sizes and poor breeding strategies could result in a loss of genetic diversity, a declining effective population size and a reduction in fitness, possibly culminating in the extinction of the breed (Kristensen et al., 2015). In addition, data driven breeding objectives in the commercial beef sector can lead to genetic erosion, which result in the failure of the population to adapt to new environmental challenges (Garrick and Golden, 2008; Molotsi et al., 2020).

This study aimed to perform a genomic characterisation of local SA cattle and sheep breeds to investigate their genetic architecture and optimal use of genomic information for their management and conservation.

## Material and Methods

### Cattle and Sheep Genotypes

Ethical approval was granted by the Ethics Committee of the Faculty of Natural and Agricultural Sciences, University of Pretoria for the use of the cattle and sheep genotypes in this study (Ethics numbers: NAS194/2020, and NAS394/2019).

Genotypes for the Beefmaster (BMA), SA Bonsmara (BON), SA Boran (BOR), Charolais (CHL), Drakensberger (DRB), SA Hereford (HFD), Nguni (NGI) and Tuli (TUL) populations were generated within the Beef Genomic Program (BGP; Walsh and Spazzoli, 2018) and made available for the study. The GeneSeek^®^ Genomic Profiler™ Bovine 150 K SNP genotyping panel, which contains 134,480 SNPs (mean SNP density: one SNP/19 kb) distributed across the 29 autosomes and two sex chromosomes, were used for genotyping. A total of 2,207 genotypes were available, ranging from 226 (TUL) to 300 per population (BMA, BON, DRB). A maximum number of 300 animals per breed was selected to avoid skewed sample sizes between breeds. Within breed, herds were selected to ensure geographic representation across South Africa.

A total of 319 genotypes representing five local South African sheep populations and three commercial breeds were made available by Grootfontein Agricultural Development Institute (GADI), and the Western Cape Agricultural Trust (WC). The indigenous genotypes included the Black Head Persian (BHP), Damara (DAM), Namaqua Afrikaner (NAM), and Pedi (PED) breeds and sample sizes ranged from 13 to 51 per population. Additionally, 16 genotypes of non-descript, fat-tailed sheep (FTT) from small holder flocks were analysed. Sixty genotypes each were included for SA Merino (MER), Dohne-Merino (DMER) and Dorper (DOR) breeds, representing commercial sheep populations. All animals were genotyped at the Agricultural Research Council’s Biotechnology Platform (ARC-BTP) using the Illumina^®^ Ovine 50 K SNP BeadChip that contains 54,241 SNPs (mean SNP density: one SNP/50.9 kb) distributed over the 26 autosomes and two sex chromosomes.

For both data sets, SNP-calling was done using the Illumina^®^ Genome Studio software v2.0 (Illumina, San Diego, California 92,122 United States). The resulting genotype input files were converted into PLINK input files using a plug-in in Genome Studio software v2.0.

### Analyses

Quality control was performed on the datasets per population using PLINK software (Purcell et al., 2007). Sample- and marker-based quality control were performed, in order to filter both non-informative SNPs and individuals from the dataset. Animals were removed on the basis of low genotyping call rates while SNPs were removed on the basis of low genotyping call rate, low mean minor allele frequency (MAF) and violation of Hardy Weinberg Equilibrium (HWE). The QC parameter thresholds were chosen to optimize animal and SNP numbers across populations. For cattle, PLINK QC parameters were set as follows: -mind 0.10, --geno 0.10, --maf 0.01 and --hwe 1 × 10^–6^; for the sheep populations, these thresholds were --mind 0.05, --geno 0.05, --maf 0.02 and --hwe 0.001.

MAF statistics, including the number of low-MAF and monomorphic SNPs, were calculated prior to any MAF-related filtering. After QC procedures, marker-based summary statistics indicating genetic diversity were estimated per population, including the mean expected and observed heterozygosity (He and Ho), MAF and LD. The *r* ^2^ measure, as proposed by Hill and Robertson (1968), was used to estimate LD. In PLINK software, no restrictions were set on the minimum *r* ^2^ (--ld-window-r2 0) and inter-SNP distance (--ld-window-kb 99,999) allowed for LD estimation. The effective population size (Ne) of each population was calculated using the LD-based SNeP software (Barbato et al., 2015).

Both the sheep and cattle datasets were merged in PLINK (Purcell et al., 2007). After QC on the merged data sets, LD pruning was additionally applied before principal component and admixture analysis, using the PLINK parameters --indep-pairwise 50 5 0.5. Subsets of 76,932 and 45,114 SNPs were retained for cattle and sheep, respectively, and applied in subsequent analyses. SNP-based genetic relatedness between individuals was calculated using GCTA version 1.24 (Genome-wide Complex Trait Analysis) (Yang et al., 2011). A genetic relationship matrix was created, followed by the estimation of eigenvalues and eigenvectors for the first three principal components, after which R (Paradis et al., 2004) was used to plot the PCA plots for both sheep and cattle populations.

Default settings in ADMIXTURE 1.23 software (Alexander et al., 2009) was used to determine the genetic population structure of both the sheep and cattle populations, through the maximum likelihood estimation of ancestry. The most likely K-value for plot visualisation was determined based on the lowest cross-validation error estimate. Thereafter bar plots were generated to visualize the inferred ancestral clusters for each K-value, by using Genesis version 0.2.3 software (Buchmann and Hazelhurst, 2014; University of the Witwatersrand, Johannesburg, SA, http://www.bioinf.wits.ac.za/software/genesis).

Inbreeding levels in both sheep and cattle populations were investigated by means of both SNP and runs of homozygosity-based coefficients (F_IS_ and F_ROH_, respectively) using PLINK (Purcell et al., 2007). The F_ROH_ coefficients were derived by performing the following calculation proposed by McQuillan et al. (2008):

$$F_{ROH}=\;\frac{\Sigma L_{ROH}}{\Sigma L_{AUTO}}$$

where: L_ROH_ = the length of ROH in one individual.

L_AUTO_ = the length of the genome covered by SNPs, excluding the centromeres.

Descriptive statistics on the number and length characteristics of ROH were furthermore reported. The proportion of segments within predefined lengths of one to 3.99 Mb, 4–7.99 Mb, 8–11.99 Mb, 12–15.99 Mb and ≥16 Mb were calculated. For ROH identification, default PLINK settings were predominantly used; however, for the cattle populations the minimum SNP density was set to one SNP/25 kb and the maximum gap to 500 kb, allowing zero opposing genotypes and two missing genotypes within a sliding window.

The --fst -within option implemented in PLINK was used to estimate Wright’s F_ST_ values to determine pairwise genetic differentiation among breeds. The methodology introduced by Weir and Cockerham (1984) is applied when this PLINK option is executed. The F_ST_ values can range from 0 to 1, where low F_ST_ values among subpopulations indicate a low level of genetic divergence in the population, whereas a value of 0 indicates that there is no subdivision between the populations.

The estimated pairwise F_ST_ values were used to determine the genetic distance between populations in order to construct a phylogenetic tree. The phylogenetic tree was created and visualized using the APE package in R software (Paradis et al., 2004).

## Results

### Mean Minor Allele Frequency Distribution

**FIGURE 1:**
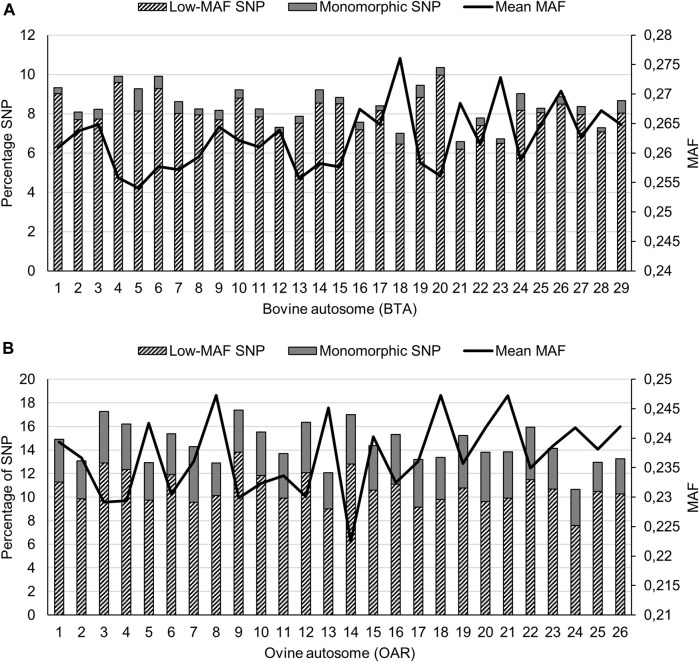
Minor allele frequency (MAF) statistics across cattle **(A)**, and sheep **(B)** populations.

The mean (±standard deviation) MAF across all cattle populations was 0.308 (±0.123), with the lowest within-population mean ranging from 0.220 (±0.151) for BOR to 0.316 (±0.120) for BMA. The NGI and TUL populations had the second and third lowest mean MAF with values of 0.249 (±0.148) and 0.258 (±0.144), respectively. The inclusion of *Bos taurus* breeds, and the composites that contain significant proportions of *Bos taurus* ancestry, inflated the estimation of low-MAF statistics and therefore the chromosome-wide MAF statistics are only reported for non-discovery breeds in the bottom half of the MAF spectrum (Figure 1A). The highest mean MAF was observed on BTA18, and the lowest on BTA5. Autosome BTA1 harboured the most SNPs (714 SNPs) with low MAF (<0.05), whereas BTA5 harboured the highest percentage of monomorphic SNPs (1.13%).

The across-population mean MAF (±standard deviation) for the sheep populations was estimated at 0.275 (±0.138). Of the indigenous sheep populations, the lowest within-population mean (0.243) was observed for the BHP population. The mean MAF estimates of the commercial DMER, DOR and MER were 0.267, 0.250, 0.271. Across the indigenous populations (Figure 1B), the highest autosome-wide mean MAF was observed for OAR18 (0.247), whilst the lowest value was for OAR14 (0.222). The highest percentage of low-MAF SNPs, defined as MAF< 0.05, was observed on OAR9 (13.8%) even though OAR7 contained the highest percentage of monomorphic SNPs (4.71%).

### Linkage Disequilibrium and LD-Based Effective Population Sizes

For both species, the LD (*r* ^2^) was generally weak when the limit of genetic distance between pairwise SNP comparisons were set to 1 Mb (i.e. larger distances between SNPs were allowed), but increased when a more stringent limitation was applied on the inter-SNP distance to consider (i.e., 100 kb and 50 kb). For cattle, the mean within-population LD estimates (considering SNPs separated by ≤100 kb) ranged from 0.165 for BMA to 0.270 for the HFD population. Within the Sanga subspecies, the mean *r* ^2^ values were 0.189, 0.173, and 0.192 for the DRB, NGI and TUL populations, respectively, whereas the indicine BOR population had a mean value of 0.185. For sheep, these values ranged from 0.186 (PED) to 0.309 (FTT) for the indigenous populations and from 0.147 (DMER) to 0.180 (DOR) for the commercial populations.

The mean chromosome-wide LD ranged from 0.114 (BTA28) to 0.189 (BTA14) for Sanga and indicine cattle (Supplementary Figure 1A), and 0.114 (OAR20) to 0.165 (OAR3) for non-commercial, indigenous sheep populations when SNPs were separated by ≤100 kb (Supplementary Figure 1B). The mean autosome-wide SNP density remained relatively constant across the cattle populations, except for a few autosomes that were more densely populated (e.g., BTA14, BTA20, and BTA24). The afore-mentioned cattle autosomes also displayed stronger autosome-wide LD (e.g., BTA14: mean SNP density = 16.23kb/SNP, and mean LD = 0.189). Across the sheep populations, the SNP density was more variable (Supplementary Figure 1B). A similar trend of weaker LD for more sparsely populated autosomes was observed. The least densely populated ovine autosome, OAR23 (74.32kb/SNP), displayed the second lowest mean LD (0.129).

The effective population size (Ne) of all cattle and sheep populations showed a decrease over generations, as expected (Supplementary Figure S2). The NGI population showed the smallest decline in LD-based Ne over time, with an estimated Ne of 421 approximately 13 generations ago. The TUL has the smallest current Ne, estimated at 147 individuals. The estimated Ne for the sheep populations were markedly smaller than estimated for cattle, ranging from 35 for BHP to 190 for the MER population 13 generations ago. The sensitivity of Ne to smaller samples sizes should be considered when interpreting these results.

### Genetic Diversity and Inbreeding Coefficients

Across the cattle populations, the average observed heterozygosity (Ho) level was 0.362, ranging from 0.322 (He = 0.318) for BOR to 0.404 (He = 0.404) for BMA. The mean Ho level across the sheep populations was slightly higher than for most cattle populations, with population-wide Ho values ranging from 0.327 (He = 0.336) for the FTT population to 0.367 (He = 0.363) for the DMER population (Table 1). Both F_IS_ and F_ROH_ inbreeding coefficients were generally not significantly different from zero, and served as a validation of the respective losses (or gains) indicated by the heterozygosity rates. Across species, the cattle populations displayed slightly higher estimates of the F_ROH_ inbreeding coefficient compared to the sheep populations. There was a tendency towards higher F_ROH_ estimates for commercial populations, (e.g., HFD cattle and DOR sheep).

**TABLE 1:** Summary statistics of the genetic diversity and inbreeding coefficients within several cattle and sheep populations occurring in South Africa.

Breed	n	He	Ho	F_ROH_	F_IS_
Cattle					
BMA	300	0.404	0.404	0.008	0.3 × 10^−3^
BON	300	0.366	0.366	0.020	0.9 × 10^−3^
BOR	270	0.318	0.322	0.007	^−^0.013
CHL	278	0.380	0.381	0.015	^−^0.002
DRB	300	0.364	0.360	0.026	0.011
HFD	241	0.376	0.375	0.029	0.002
NGI	292	0.338	0.338	0.006	0.001
TUL	215	0.349	0.352	0.022	^−^0.009
**Sheep**					
BHP	13	0.328	0.353	-	^−^0.077
DAM	30	0.335	0.339	0.004	^−^0.013
DOR	60	0.348	0.336	0.031	0.036
DMER	60	0.363	0.367	0.011	^−^0.011
FTT	16	0.336	0.327	0.008	0.026
MER	60	0.366	0.362	0.011	0.009
NAM	51	0.344	0.337	0.002	0.021
PED	29	0.374	0.356	0.004	0.049

Cattle: BMA = Beefmaster, BON = Bonsmara, BOR = Boran, CHL = Charolais, DRB = Drakensberger, HFD = Hereford, NGI = Nguni, TUL = Tuli; Sheep: BHP = Black Headed Persian, DAM = Damara, DOR = Dorper, DMER = Dohne Merino, FTT = Fat-tail, MER = Merino, NAM = Namaqua Afrikaner, PED = Pedi.

### Runs of Homozygosity

**FIGURE 2:**
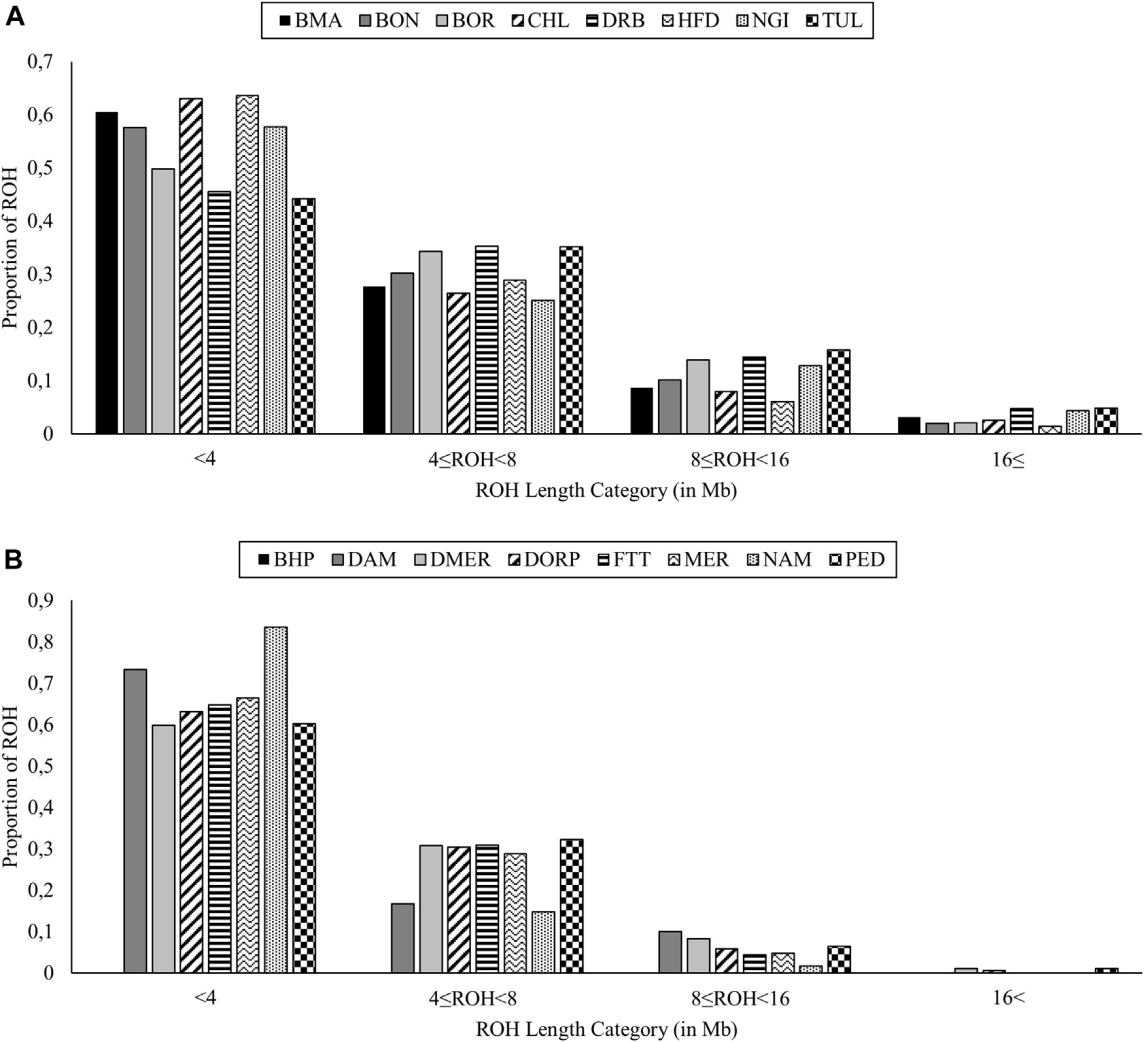
Distribution of runs of homozygosity (ROH) segments within different length categories for cattle **(A)**, and sheep **(B)** populations.

The ROH analyses identified 17,362 autozygous segments in total across the eight cattle populations. The mean number of ROH per population ranged from 733 (NGI) to 4,069 (HFD) and the mean ROH length ranged from 4.273 Mb (HFD) to 6.065 Mb (TUL). For all cattle populations, the highest proportion of ROH were observed within the shortest length category (<4 Mb) and these proportions ranged from 0.442 (TUL) to 0.636 (HFD) (Figure 2A). Within the largest ROH length category (>16 Mb), the TUL ranked the highest (proportion units = 0.049) and the HFD lowest (proportion units = 0.015).

A smaller total of 1,537 ROH segments were identified in sheep populations, with the bulk of these segments identified in the commercial populations (DOR = 742, DMER = 374 and MER = 170). Within the indigenous group, no ROH segments were identified for the BHP population whereas the highest number of ROH segments were identified for the PED population (*n* = 91) (Figure 2B). The vast majority of ROH segments fell within the shortest length category; within this category, the DMER population had the lowest proportion (0.599). The DMER, DOR and PED populations were the only populations with ROH segments exceeding 16 Mb in length.

### Population Structure

**FIGURE 3:**
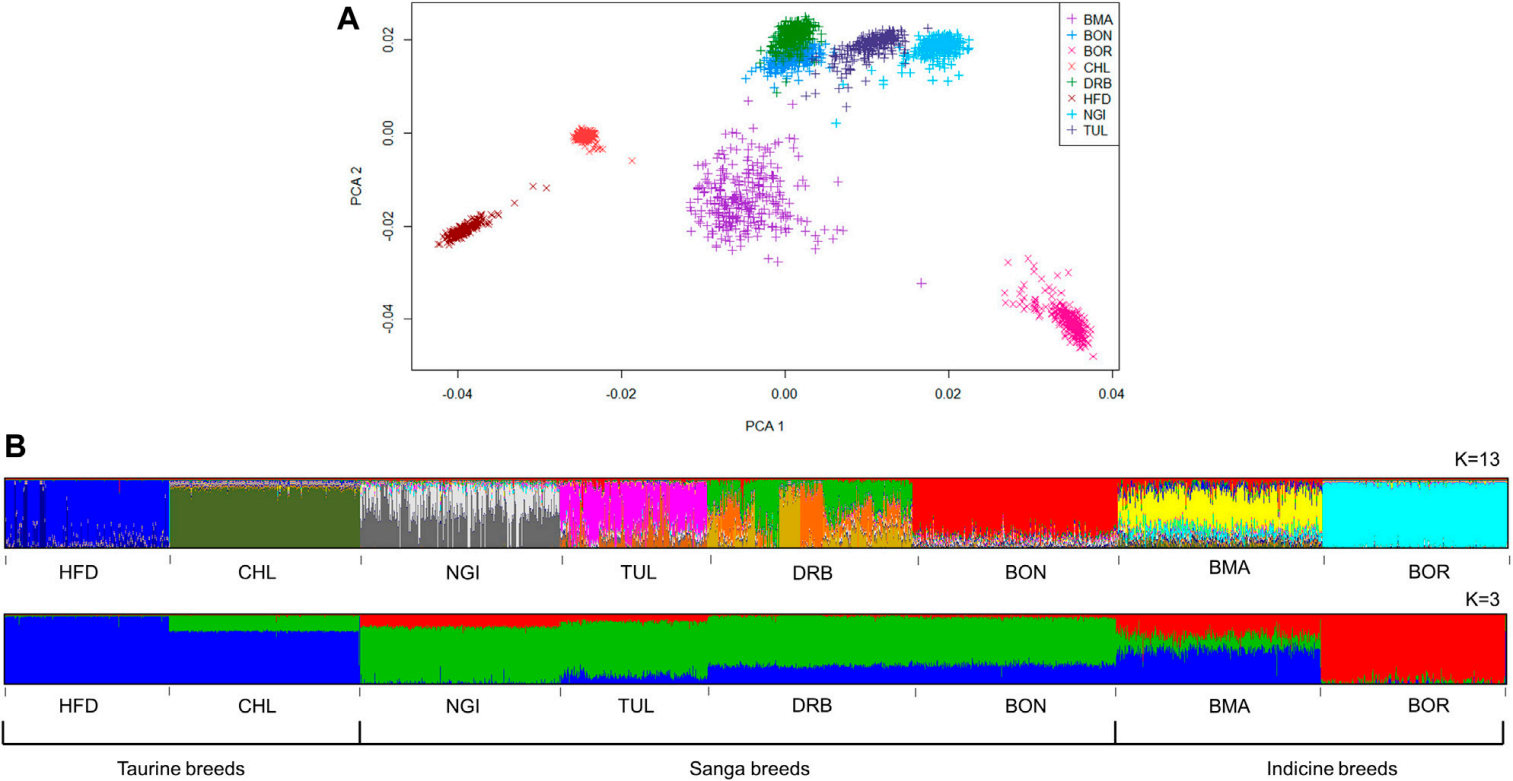
Genetic structure of South African cattle breeds, according to model-based clustering **(A)** and principle component analysis **(B)**.

The first and second principal components (PC1 and PC2) explained 12.32% of the total variation between cattle populations, and separated them into distinct clusters (Figure 3A). The Sanga, and Sanga-derived composite (BON), were clustered together, whilst the taurine (with CHL and HFD in close proximity to one another), and the indicine (BOR) clustered on opposite extremes of this subspecies. The BMA cluster was central to, but separate from, the indicine, Sanga and taurine clusters with some animals showing close relatedness to the Sanga group.

**FIGURE 4:**
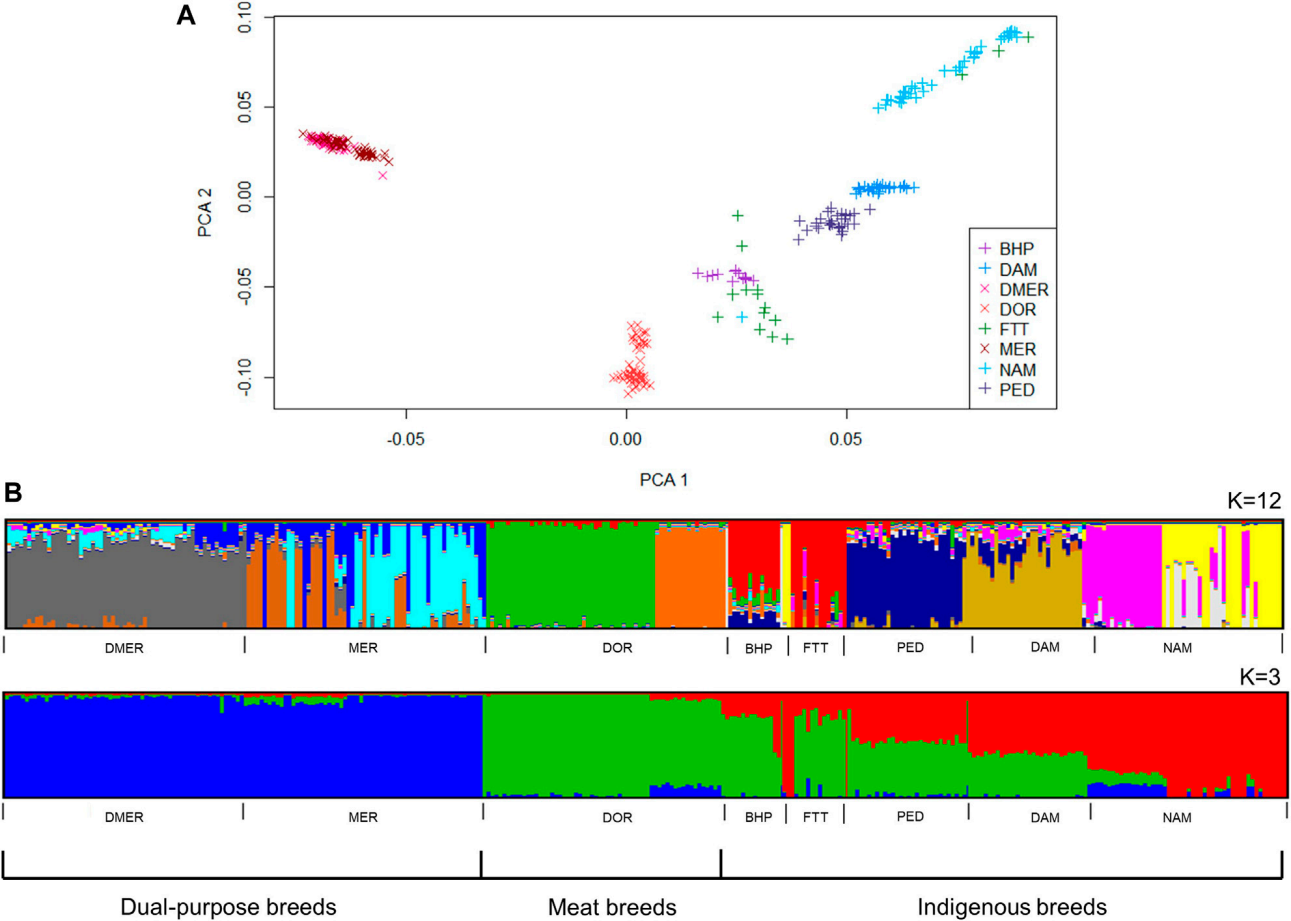
Genetic structure of South African sheep breeds, according to model-based clustering **(A)** and principle component analysis **(B)**.

PC1 and PC2 explained 13.3% of the total variation between the sheep populations. The BHP, DAM, NAM and PED populations formed distinct clusters, while the cluster for non-descript fat tailed sheep were less well defined and FTT animals were observed within the fat-tailed NAM population and the fat-rumped BHP clusters (Figure 4A). Of the commercial breeds, the DOR population formed a distinct cluster in closer proximity to the indigenous breeds, whereas the DMER and the MER populations formed a single overlapping cluster on the extreme end of the PCA plot.

The most likely K values for the cattle and sheep populations were estimated as 13 and 12, respectively. At K = 3, subdivision by ancestral clusters (*Bos indicus*, *Bos taurus* and *Bos taurus africanus*) was observed, with composites displaying clear influences from the ancestral cluster that their base breeds belong to. Interestingly, the DRB breed displayed similar proportions of taurine ancestry to the BON breed, with smaller proportions of indicine ancestry than NGI and TUL breeds (Figure 3B). At K = 13, population substructure within the DRB, HFD, NGI, and TUL was evidenced by increased admixture. The MER population showed the highest level of admixture of the sheep populations (Figure 4B). The FTT individuals showed a clear shared ancestry with BHP, while the NAM and MER showed distinct population substructure with three sub-groupings each.

**FIGURE 5:**
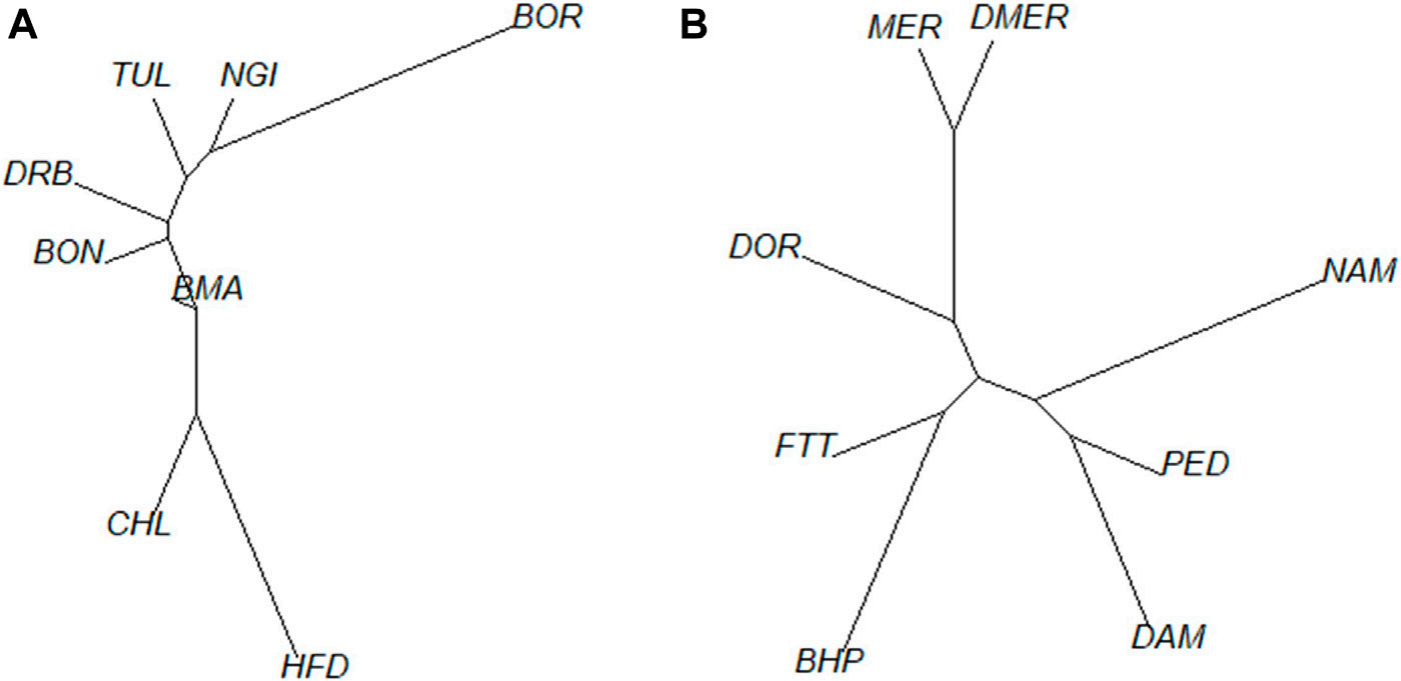
Genetic distance between populations based on pairwise F_ST_ estimates for cattle **(A)** and sheep **(B)**.

The phylogenetic tree corroborated the results of the ADMIXTURE plots (Figure 5A). The phylogeny illustrated divergence of the BOR breed (predominantly *Bos indicus*) from CHL and HFD breeds (European *Bos taurus*), with Sanga breeds (BON, DRB, NGI and TUL) being intermediate. The BMA branch was closer to the European *Bos taurus* group. Figure 5B showed close relatedness between DMER and MER sheep populations, with divergence of these and the DOR population (a commercial breed) from indigenous populations. Within the indigenous group, the FTT showed close relatedness to BHP while DAM and PED were phylogenetically less differentiated.

## Discussion

A number of studies on indigenous populations have reported ascertainment bias, generally evidenced by low genome-wide MAF, and this can be attributed to their exclusion from the initial development of the commercial genotyping panels applied. The MAF values observed in the current study (NGI: 0.249 to DRB: 0.274) confirmed the ascertainment bias previously reported in indigenous SA beef cattle (Qwabe et al., 2013; Zwane et al., 2016; Lashmar et al., 2018). The MAF values for the exotic breeds that were included, (CHL: 0.280 and HFD: 0.275) were similar to those reported in other studies (He et al., 2018). Likewise, the limited inclusion of loci representing indigenous breeds in international ovine SNP chips have introduced systematic bias in estimates of variation within and between sheep populations (Morin et al., 2004). The high levels of low-MAF SNPs (mean = 10.72%) and monomorphic SNPs (mean = 3.71%) observed for indigenous and African fat-tailed breeds, are probably a reflection of their underrepresentation in the development of the Ovine SNP50 BeadChip. The ascertainment bias might be less severe for the cattle populations sampled, as these breeds carry varying proportions of European taurine ancestry.

The results for observed levels of heterozygosity indicate moderate genetic diversity for the majority of beef cattle populations included in the study (BOR: 0.322 to BMA: 0.404). These levels are similar to those reported in a recent study on 53 cattle breeds (e.g., HFD: 0.331 and CHL: 0.345; Zhang et al., 2018). The BMA had the highest level of diversity (0.404) which can be attributed to its composite nature and as it is a relatively young breed that was only introduced to SA in 1986. The heterozygosity levels for the sheep populations were moderate with slight differences and were also comparable to previous studies (Sandenbergh et al., 2016; Molotsi et al., 2017). Available literature indicates relatively small population sizes for these indigenous sheep breeds with less than 1,000 breeding females for NAM (Qwabe et al., 2013) and PED (FAO-DAD-IS, 2021). However, consensus data on SA indigenous sheep numbers are limited as they are mostly found in small holder systems with no access to performance recording. The moderate genomic diversity reported here for these sheep populations is therefore encouraging for designing conservation strategies. As a result of the limited numbers and less intense artificial selection in comparison to commercial breeds, these breeds will, however, be more likely to experience evolutionary constraints, genetic drift, and inbreeding (Kristensen et al., 2015).

The majority of ROH for all cattle breeds were observed in the shortest category (<4 Mb). ROH of 1 Mb in size has been associated with more distant inbreeding of up to 50 generations in age and segments of 4 Mb in length to 12.5 generations in age, equating to approximately 75 years (Mastrangelo et al., 2017; 2020). The ROH profiles of the cattle populations in this study is therefore indicative of more distant ancestral effects. Although the frequency of long ROH is currently low (range of ROH>16 Mb: 1.45% in HFD to 4.88% in TUL), caution should be taken when pipelines for genomic selection (GS) are implemented for these breeds. International studies have indicated an increase in the frequency of long ROH as a result of GS, and these ROH segments have proven to enhance deleterious variation (Szpiech et al., 2013). The F_ROH_ and F_IS_ estimates were not significantly different from zero, supporting low inbreeding levels despite selection for specific traits, and this concurred with estimates from a meta-analysis (F_ROH_ range: 0.01–0.07) of ROH that included 151 global populations (Mastrangelo et al., 2020).

In the sheep populations a relatively low number of ROH segments were identified per indigenous population, ranging from as few as 30 for the DAM breed to 93 for PED and this was consequently supported by low positive estimates of F_ROH_ (and F_IS_). The number of identified ROH segments is expected to grow with the addition of more genotyped animals, and a higher density of population-specific SNPs (ideally, through whole-genome sequencing efforts). The longer ROH observed in the PED is supported by the history of the breed, as until the 1980s the breed was solely kept in rural communities, which is not a conducive environment for implementation of selection and mating practises (Mavule et al., 2013). Larger numbers of genotypes will be required for a more accurate comparison of ROH parameters between indigenous and commercial breeds.

Low LD levels for indigenous cattle populations could result from ascertainment bias, and the consequent removal of more low-MAF SNPs (Makina et al., 2015). On the contrary, higher LD in indigenous sheep populations (range: 0.186–0.309) can reflect their smaller population sizes, as reductions in population sizes (e.g., bottleneck events) can result in haplotype loss and subsequent increases in within-population LD levels (Slatkin, 2008). Effective population sizes for all the breeds followed similar decreasing trends of other studies on cattle and sheep breeds (Gasca-Pineda et al., 2013; Makina et al., 2015; Prieur et al., 2017). The effective population sizes observed for sheep were lower compared to cattle, even though the cattle populations have been subjected to more intense selection. Although a reduced Ne may indicate lower genetic variation, it does not necessarily correspond with the genetic variation and inbreeding estimates and could be an artefact of lower sample sizes, which could in turn influence the degree of LD captured. Other studies on cattle, for example, have reported comparably smaller Ne estimates, however, using significantly smaller populations sizes for estimation (range: 10–51; Senczuk et al., 2021). Recommendations for a minimum Ne in domestic breeds including cattle and sheep were set as 50 to avoid inbreeding on the short term and 500 on the long term (Leroy et al., 2013).

The local SA cattle breeds are recognised as commercial breeds, and have the advantage of monitored inbreeding using pedigrees and performance recordings. There is however variation in participation in animal recording with reported pedigree completeness over six generations varying from 28% for Boran, 38% for Nguni compared to 70% for Drakensberger cattle (Abin et al., 2016). The lack of pedigree depth for both local cattle and sheep populations may compromise the accurate estimation of inbreeding. Based on simulated data, Wang (2016) confirmed the importance of a complete pedigree to estimate accurate pedigree-based inbreeding. Inbreeding parameters based on genomic information, such as F_ROH_ are not adversely affected by the allele frequencies and pedigree errors (Zhang et al., 2015) and more useful for managing inbreeding.

The principal component analysis (PCA) and model-based admixture inferences both illustrated a clear distinction between the Sanga breeds, the composites and the *Bos Taurus* breeds. Furthermore, a distinction was observed within the Sanga component, with a separation between the DRB and the other two Sanga breeds (NGI, and TUL). In both instances, the differentiation can be explained by differences in genetic composition. Much like Sanga cattle, the BOR breed also derives its genetic composition from three ancestral sources namely European *Bos Taurus* (EBT), African *Bos Taurus* (ABT), and *Bos indicus* (BI). However, whereas the BOR breeds is composed of higher proportions of indicine genetics (64%; Hanotte et al., 2002), Sanga cattle are predominantly taurine with varying proportions of European and African ancestry. Makina et al. (2016) indicated ABT:EBT:BI ratios of 70:30:10 and 38:46:16 for the NGI and DRB breeds, respectively. The varying proportions of African-to-European taurine ancestry can therefore explain the separation of the DRB from the other Sanga breeds.

The sheep populations exhibited distinction between the commercial and local populations based on the PCA, admixture and the pairwise F_ST_ based phylogenetic tree results. There was some overlap between the FTT and BHP populations which can be expected as the non-descript FTT animals are mostly kept in communal systems or in poorly fenced areas where admixture is more likely to occur (Molotsi et al., 2017). The animals of the NAM population formed a particularly loose cluster, indicating more variation in the genotypes of individuals of this population. This could be due to the predominant communal and smallholder systems farming systems, with lower selection pressure for specific production traits, resulting in a larger within-population variation (Kijas et al., 2009). The three distinct subgroups of NAM indicate separate lines that could be utilized in outbreeding strategies to increase genetic variation in this endangered breed to enable effective conservation strategies.

Apart from financial and infrastructural limitations, which have been widely reported (e.g., Mapiye et al., 2019; Marshall et al., 2019), there are a multitude of additional genotype-related factors to consider before genomics-based selection programs can be applied to indigenous livestock resources. Apart from taurine (ARS-UCD1.2; Rosen et al., 2020), indicine (Bos_indicus_1.0; Canavez et al., 2012) and hybrid Angus x Brahman (UOA_Brahman_1; Low et al., 2020) reference genomes, no African-specific reference genome has been published to date on the National Centre for Biotechnology Information (NCBI) database. For cattle, the pool of reference SNPs used in designing many of the commercially available genotyping panels (including the one used in the present study) are therefore only comprised of pure taurine (e.g., Matukumalli et al., 2009) or pure indicine markers (Ferraz et al., 2018). As is evidenced in this study, this poses a disadvantage given that most African breeds have heterogenous genomic architectures that are composed of a mixture between the two subspecies as well as completely unique African derived genomic signatures. Despite the sub-optimal performance of most genotyping panels, these panels have proven sufficient in estimating genomic breeding values for indigenous cattle breeds that are characterized by relatively larger population sizes and are prominent in the commercial sector (e.g., SA Bonsmara, and SA Drakensberger). Although GS is in its infancy in SA, note should be taken of the potential of GS to manage losses in genetic diversity, especially considering that these breeds are indigenous to, and adapted to, the SA producing environments.

This study shows the value of using genomic parameters to manage local and indigenous livestock populations. The general assumption that these populations are plagued by inbreeding or indiscriminate crossbreeding, was not observed in the current study. The populations included in the current study are generally in a healthy genetic condition (in terms of diversity and inbreeding levels), although they are under directional selection or part of numerically small populations. More genotypes should however be generated, especially for sheep populations, to accurately monitor diversity parameters and to exploit the unique characteristics associated with these population’s specific adaptation.

## Conclusion

This study demonstrates the applicability of genomic parameters to SA local livestock populations. Well-planned approaches, including the recording of pedigree and phenotypic information, improved mating strategies and the incorporation of genomic information could counter the challenges of directional selection in beef cattle and small population sizes of local sheep populations. Future research should be directed at investigating the genomic variation of adaptive traits for developing strategies to preserve and utilize these valuable genetic resources.

## Data Availability

The raw data supporting the conclusion of this article will be made available by the authors, without undue reservation.

## References

[B1] Abin S. Theron H. Van Marle-Köster E. (2016). Population Structure and Genetic Trends for Indigenous African Beef Cattle Breeds in South Africa. SA J. Sci. 46 (2), 152–156. 10.4314/sajas.v46i2.5

[B2] Akinmoladun O. F. Muchenje V. Fon F. N. Mpendulo C. T. (2019). Small Ruminants: Farmer's Hope in a World Threatened by Water Scarcity. Animals 9, 456. 10.3390/ani9070456 PMC6680725 31323882

[B3] Alexander D. H. Novembre J. Lange K. (2009). Fast Model-Based Estimation of Ancestry in Unrelated Individuals. Genome Res. 19, 1655–1664. 10.1101/gr.094052.109 19648217 PMC2752134

[B4] Barbato M. Orozco-terWengel P. Tapio M. Bruford M. W. (2015). SNeP: A Tool to Estimate Trends in Recent Effective Population Size Trajectories Using Genome-Wide SNP Data. Front. Genet. 6, 109. 10.3389/fgene.2015.00109 25852748 PMC4367434

[B5] Benhin J. K. (2006). Climate Change and South African Agriculture. Discussion Paper No. 21. Centre for Environmental Economics and Policy in Africa (CEEPA), University of Pretoria.

[B6] Bester J. Matjuda L. E. Rust J. M. Fourie H. J. (2003). The Nguni: A Case Study. Irene, South Africa: Animal Improvement Institute, Private Bag x2, 0062.

[B7] Buchmann R. Hazelhurst S. (2014). Genesis Manual. Available at: http://www.bioinf.wits.ac.za/software/genesis/Genesis.pdf (Accessed October 15, 2020).

[B8] Canavez F. C. Luche D. D. Stothard P. Leite K. R. M. Sousa-Canavez J. M. Plastow G. (2012). Genome Sequence and Assembly of *Bos indicus* . J. Hered. 103 (3), 342–348. 10.1093/jhered/esr153 22315242

[B9] Dzomba E. F. Chimonyo M. Snyman M. A. Muchadeyi F. C. (2020). The Genomic Architecture of South African Mutton, Pelt, Dual‐Purpose and Nondescript Sheep Breeds Relative to Global Sheep Populations. Anim. Genet. 51, 910–923. 10.1111/age.12991 32894610

[B10] FAO-DAD-IS (2021). Available online: www.fao.org/dad-is/en (Accessed May 14, 2021).

[B11] Ferraz J. B. S. Wu X. Li H. Xu J. Ferretti R. Simpson B. Walker J. Silva L. R. Garcia J. F. Tait R. G. Jr Bauck S. (2018). “Design of a Low-Density SNP Chip for *Bos indicus*: GGP Indicus Technical Characterization and Imputation Accuracy to Higher Density SNP Genotypes,” in Proceedings of the 11th World Congress of Genetics Applied to Livestock Production, Auckland, New Zealand, February 6–11, 2018, 3–7.

[B12] Garrick D. J. Golden B. L. (2008). Producing and Using Genetic Evaluations in the United States Beef Industry of Today. J. Anim. Sci. 87, E11–E18. 10.2527/jas.2008-1431 18849385

[B13] Gasca-Pineda J. Cassaigne I. Alonso R. A. Eguiarte L. E. (2013). Effective Population Size, Genetic Variation, and Their Relevance for Conservation: The Bighorn Sheep in Tiburon Island and Comparisons with Managed Artiodactyls. PLoS ONE 8 (10), e78120–22. 10.1371/journal.pone.0078120 24147115 PMC3795651

[B14] Hanotte O. Bradley D. G. Ochieng J. W. Verjee Y. Hill E. W. Rege J. E. O. (2002). African Pastoralism: Genetic Imprints of Origins and Migrations. Science 296 (5566), 336–339. 10.1126/science.1069878 11951043

[B15] He J. Guo Y. Xu J. Li H. Fuller A. Tait R. G. (2018). Comparing SNP Panels and Statistical Methods for Estimating Genomic Breed Composition of Individual Animals in Ten Cattle Breeds. BMC Genet. 19 (1), 56–14. 10.1186/s12863-018-0654-3 30092776 PMC6085684

[B16] Hill W. G. Robertson A. (1968). Linkage Disequilibrium in Finite Populations. Theoret. Appl. Genet. 38 (6), 226–231. 10.1007/bf01245622 24442307

[B17] Kijas J. W. Townley D. Dalrymple B. P. Heaton M. P. Maddox J. F. McGrath A. (2009). A Genome Wide Survey of SNP Variation Reveals the Genetic Structure of Sheep Breeds. PLoS ONE 4 (3), e4668–13. 10.1371/journal.pone.0004668 19270757 PMC2652362

[B18] Kristensen T. N. Hoffmann A. A. Pertoldi C. Stronen A. V. (2015). What Can Livestock Breeders Learn from Conservation Genetics and *Vice Versa*? Front. Genet. 6, 38. 10.3389/fgene.2015.00038 25713584 PMC4322732

[B19] Kunene N. W. Bezuidenhout C. C. Nsahlai I. V. (2009). Genetic and Phenotypic Diversity in Zulu Sheep Populations: Implications for Exploitation and Conservation. Small Ruminant Res. 84, 100–107. 10.1016/j.smallrumres.2009.06.012

[B20] Kunene N. W. Bezuidenhout C. C. Nsahlai I. V. Nesamvuni E. A. (2011). A Review of Some Characteristics, Socio-Economic Aspects and Utilization of Zulu Sheep: Implications for Conservation. Trop. Anim. Health Prod. 43, 1075–1079. 10.1007/s11250-011-9823-3 21509458

[B21] Kunene-Ngubane P. E. (2015). Identification of Opportunities for Organic Beef Production from Nguni Cattle to Enhance Food Security by Communal Farmers in KwaZulu-Natal South Africa. PhD Dissertation. Pietermaritzburg, South Africa: University of KwaZulu-Natal.

[B22] Lashmar S. F. Visser C. van Marle-Köster E. Muchadeyi F. C. (2018). Genomic Diversity and Autozygosity within the SA Drakensberger Beef Cattle Breed. Livestock Sci. 212, 111–119. 10.1016/j.livsci.2018.04.006

[B23] Leroy G. Mary-Huard T. Verrier E. Danvy S. Charvolin E. Danchin-Burge C. (2013). Methods to Estimate Effective Population Size Using Pedigree Data: Examples in Dog, Sheep, Cattle and Horse. Genet. Sel. Evol. 45 (1), 1–10. 10.1186/1297-9686-45-1 23281913 PMC3599586

[B24] Low W. Y. Tearle R. Liu R. Koren S. Rhie A. Bickhart D. M. (2020). Haplotype-Resolved Genomes Provide Insights into Structural Variation and Gene Content in Angus and Brahman Cattle. Nat. Commun. 11 (1), 2071. 10.1038/s41467-020-15848-y 32350247 PMC7190621

[B25] Madilindi M. A. Banga C. B. Bhebhe E. Sanarana Y. P. Nxumalo K. S. Taela M. G. (2020). Genetic Diversity and Relationships Among Three Southern African Nguni Cattle Populations. Trop. Anim. Health Prod. 52 (2), 753–762. 10.1007/s11250-019-02066-y 31529304

[B26] Makina S. O. Whitacre L. K. Decker J. E. Taylor J. F. MacNeil M. D. Scholtz M. M. (2016). Insight into the Genetic Composition of South African Sanga Cattle Using SNP Data from Cattle Breeds Worldwide. Genet. Sel. Evol. 48 (1), 88. 10.1186/s12711-016-0266-1 27846793 PMC5111355

[B27] Makina S. O. Taylor J. F. van Marle-Köster E. Muchadeyi F. C. Makgahlela M. L. MacNeil M. D. (2015). Extent of Linkage Disequilibrium and Effective Population Size in Four South African Sanga Cattle Breeds. Front. Genet. 6, 337. 10.3389/fgene.2015.00337 26648975 PMC4664654

[B28] Mapiye C. Chikwanha O. C. Chimonyo M. Dzama K. (2019). Strategies for Sustainable Use of Indigenous Cattle Genetic Resources in Southern Africa. Diversity 11, 214. 10.3390/d11110214

[B29] Marshall K. Gibson J. P. Mwai O. Mwacharo J. M. Haile A. Getachew T. (2019). Livestock Genomics for Developing Countries - African Examples in Practice. Front. Genet. 10, 297. 10.3389/fgene.2019.00297 31105735 PMC6491883

[B30] Mastrangelo S. Tolone M. Ben Jemaa S. Sottile G. Di Gerlando R. Cortés O. (2020). Refining the Genetic Structure and Relationships of European Cattle Breeds through Meta-Analysis of Worldwide Genomic SNP Data, Focusing on Italian Cattle. Sci. Rep. 10 (1), 14522. 10.1038/s41598-020-71375-2 32883980 PMC7471305

[B31] Mastrangelo S. Portolano B. Di Gerlando R. Ciampolini R. Tolone M. Sardina M. T. (2017). Genome-Wide Analysis in Endangered Populations: A Case Study in Barbaresca Sheep. Animal 11 (7), 1107–1116. 10.1017/s1751731116002780 28077191

[B32] Matukumalli L. K. Lawley C. T. Schnabel R. D. Taylor J. F. Allan M. F. Heaton M. P. (2009). Development and Characterization of a High Density SNP Genotyping Assay for Cattle. PloS one 4 (4), e5350. 10.1371/journal.pone.0005350 19390634 PMC2669730

[B33] Mavule B. S. Muchenje V. Kunene N. W. (2013). Characterization of Zulu Sheep Production System: Implications for Conservation and Improvement. Scientific Res. essays 8 (26), 1226–1238. 10.5897/SRE2013.1872

[B34] McQuillan R. Leutenegger A.-L. Abdel-Rahman R. Franklin C. S. Pericic M. Barac-Lauc L. (2008). Runs of Homozygosity in European Populations. Am. J. Hum. Genet. 83 (3), 359–372. 10.1016/j.ajhg.2008.08.007 18760389 PMC2556426

[B35] Molotsi A. H. Dube B. Cloete S. W. P. (2020). The Current Status of the Indigenous Ovine Genetic Resources in Southern Africa and Future Sustainable Utilisation to Improve Livelihoods. Diversity 12, 14. 10.3390/d12010014

[B36] Molotsi A. H. Taylor J. F. Cloete S. W. P. Muchadeyi F. Decker J. E. Whitacre L. K. (2017). Genetic Diversity and Population Structure of South African Smallholder Farmer Sheep Breeds Determined Using the OvineSNP50 Beadchip. Trop. Anim. Health Prod. 49, 1771–1777. 10.1007/s11250-017-1392-7 28916880

[B37] Morin P. A. Luikart G. Wayne R. K. the SNP workshop group (2004). SNPs in Ecology, Evolution and Conservation. Trends Ecol. Evol. 19 (4), 208–216. 10.1016/j.tree.2004.01.009

[B38] Mwai O. Hanotte O. Kwon Y.-J. Cho S. (2015). - Invited Review - African Indigenous Cattle: Unique Genetic Resources in a Rapidly Changing World. Asian Australas. J. Anim. Sci. 28 (7), 911–921. 10.5713/ajas.15.0002r 26104394 PMC4478499

[B39] Nedambale T. L. Mapholi N. O. Sebei J. P. O’Neill H. A. Nxumalo K. S. Nephawe K. A. (2020). Assessment of Genetic Variation in Bapedi Sheep Using Microsatellite Markers. S. Afr. J. Anim. Sci. 50 (2), 318–324. 10.4314/sajas.v50i2.15

[B40] Paradis E. Claude J. Strimmer K. (2004). APE: Analyses of Phylogenetics and Evolution in R Language. Bioinformatics 20 (2), 289–290. 10.1093/bioinformatics/btg412 14734327

[B41] Prieur V. Clarke S. M. Brito L. F. McEwan J. C. Lee M. A. Brauning R. (2017). Estimation of Linkage Disequilibrium and Effective Population Size in New Zealand Sheep Using Three Different Methods to Create Genetic Maps. BMC Genet. 18 (1), 68–19. 10.1186/s12863-017-0534-2 28732466 PMC5521107

[B42] Purcell S. Neale B. Todd-Brown K. Thomas L. Ferreira M. A. R. Bender D. (2007). PLINK: A Tool Set for Whole-Genome Association and Population-Based Linkage Analyses. Am. J. Hum. Genet. 81 (3), 559–575. Available at: https://linkinghub.elsevier.com/retrieve/pii/S0002929707613524 . 10.1086/519795 17701901 PMC1950838

[B43] Qwabe S. O. van Marle-Köster E. Visser C. (2013). Genetic Diversity and Population Structure of the Endangered Namaqua Afrikaner Sheep. Trop. Anim. Health Prod. 45, 511–516. 10.1007/s11250-012-0250-x 22930466

[B44] Rosen B. D. Bickhart D. M. Schnabel R. D. Koren S. Elsik C. G. Tseng E. (2020). De Novo assembly of the Cattle Reference Genome with Single-Molecule Sequencing. Gigascience 9 (3), giaa021. 10.1093/gigascience/giaa021 32191811 PMC7081964

[B45] Rust J. M. Rust T. (2013). Climate Change and Livestock Production: A Review with Emphasis on Africa. S. Afr. J. Anim. Sci. 43 (3), 256–267. 10.4314/sajas.v43i3.3

[B46] Sanarana Y. Visser C. Bosman L. Nephawe K. Maiwashe A. van Marle-Köster E. (2016). Genetic Diversity in South African Nguni Cattle Ecotypes Based on Microsatellite Markers. Trop. Anim. Health Prod. 48, 379–385. 10.1007/s11250-015-0962-9 26611262

[B47] Sandenbergh L. Cloete S. W. P. Roodt-Wilding R. Snyman M. A. Bester-van der Merwe A. E. (2016). Evaluation of the ovineSNP50 Chip for Use in Four South African Sheep Breeds. S. Afr. J. Anim. Sci. 46 (1), 90–93. 10.4314/sajas.v46i1.11

[B48] Senczuk G. Mastrangelo S. Ajmone-Marsan P. Becskei Z. Colangelo P. Colli L. (2021). On the Origin and Diversification of Podolian Cattle Breeds: Testing Scenarios of European Colonization Using Genome-Wide SNP Data. Genet. Sel. Evol. 53 (1), 48–16. 10.1186/s12711-021-00639-w 34078254 PMC8173809

[B49] Slatkin M. (2008). Linkage Disequilibrium - Understanding the Evolutionary Past and Mapping the Medical Future. Nat. Rev. Genet. 9 (6), 477–485. 10.1038/nrg2361 18427557 PMC5124487

[B50] Soma P. Kotze A. Grobler J. P. van Wyk J. B. (2012). South African Sheep Breeds: Population Genetic Structure and Conservation Implications. Small Ruminant Res. 103 (2–3), 112–119. 10.1016/j.smallrumres.2011.09.041

[B51] Stringer L. C. Fraser E. D. G. Harris D. Lyon C. Pereira L. Ward C. F. M. (2020). Adaptation and Development Pathways for Different Types of Farmers. Environ. Sci. Pol. 104, 174–189. 10.1016/j.envsci.2019.10.007

[B52] Szpiech Z. A. Xu J. Pemberton T. J. Peng W. Zöllner S. Rosenberg N. A. (2013). Long Runs of Homozygosity Are Enriched for Deleterious Variation. Am. J. Hum. Genet. 93 (1), 90–102. 10.1016/j.ajhg.2013.05.003 23746547 PMC3710769

[B53] Tuli Cattle (2021). Tuli Cattle Breeder’s Society of South Africa. Available at: http://www.tulicattle.co.za/p2/history/the-tuli-cattle-story.html (Accessed May 17, 2021).

[B54] Van der Westhuizen L. MacNeil M. D. Scholtz M. M. Neser F. W. C. Makgahlela M. L. Van Wyk J. B. (2020). Genetic Variability and Relationships in Nine South African Cattle Breeds Using Microsatellite Markers. Trop. Anim. Health Prod. 52, 177–184. 10.1007/s11250-019-02003-z 31388877

[B55] van Marle-Köster E. Visser C. (2018). Genetic Improvement in South African Livestock: Can Genomics Bridge the Gap Between the Developed and Developing Sectors? Front. Genet. 9, 331. 10.3389/fgene.2018.00331 30190725 PMC6115519

[B56] Walsh K. Spazzoli R. (2018). Assessing the Economic Impact of the South African Beef Genomics Programme. Available at: https://www.novaeconomics.co.za/our-work/705-2 (Accessed May 17, 2021).

[B57] Wang J. (2016). Pedigrees or Markers: Which Are Better in Estimating Relatedness and Inbreeding Coefficient? Theor. Popul. Biol. 107, 4–13. 10.1016/j.tpb.2015.08.006 26344786

[B58] Wilson R. T. (2011). Populations and Production of Fat-Tailed and Fat-Rumped Sheep in the Horn of Africa. Trop. Anim. Health Prod. 43, 1419–1425. 10.1007/s11250-011-9870-9 21516441

[B59] Yang J. Lee S. H. Goddard M. E. Visscher P. M. (2011). GCTA: A Tool for Genome-Wide Complex Trait Analysis. Am. J. Hum. Genet. 88 (1), 76–82. 10.1016/j.ajhg.2010.11.011 21167468 PMC3014363

[B60] Zhang M. Peng W. F. Hu X. J. Zhao Y. X. Lv F. H. Yang J. (2018). Global Genomic Diversity and Conservation Priorities for Domestic Animals Are Associated with the Economies of Their Regions of Origin. Sci. Rep. 8 (1), 11677. 10.1038/s41598-018-30061-0 30076315 PMC6076285

[B61] Zhang Q. Calus M. P. Guldbrandtsen B. Lund M. S. Sahana G. (2015). Estimation of Inbreeding Using Pedigree, 50k SNP Chip Genotypes and Full Sequence Data in Three Cattle Breeds. BMC Genet. 16 (1), 88–11. 10.1186/s12863-015-0227-7 26195126 PMC4509611

[B62] Zwane A. A. Maiwashe A. Makgahlela M. L. Choudhury A. Taylor J. F. van Marle-Köster E. (2016). Genome-Wide Identification of Breed-Informative Single-Nucleotide Polymorphisms in Three South African Indigenous Cattle Breeds. S. Afr. J. Anim. Sci. 46 (3), 302–312. 10.4314/sajas.v46i3.10

