# Supplementary figures and images for: Whole-Genome SNP Characterisation Provides Insight for Sustainable Use of Local South African Livestock Populations
Source: Front Genet. 2021 Oct 28;12:714194. doi: 10.3389/fgene.2021.714194 (PMC8581043; doi:10.3389/fgene.2021.714194)

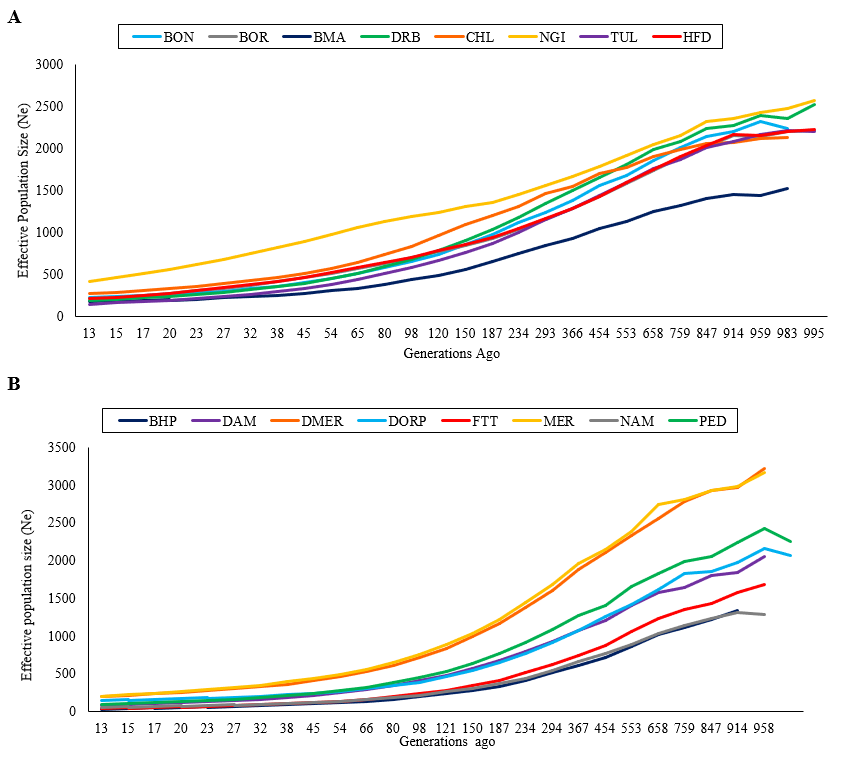

Supplement: Supplementary file 1 [file Image3.TIF]

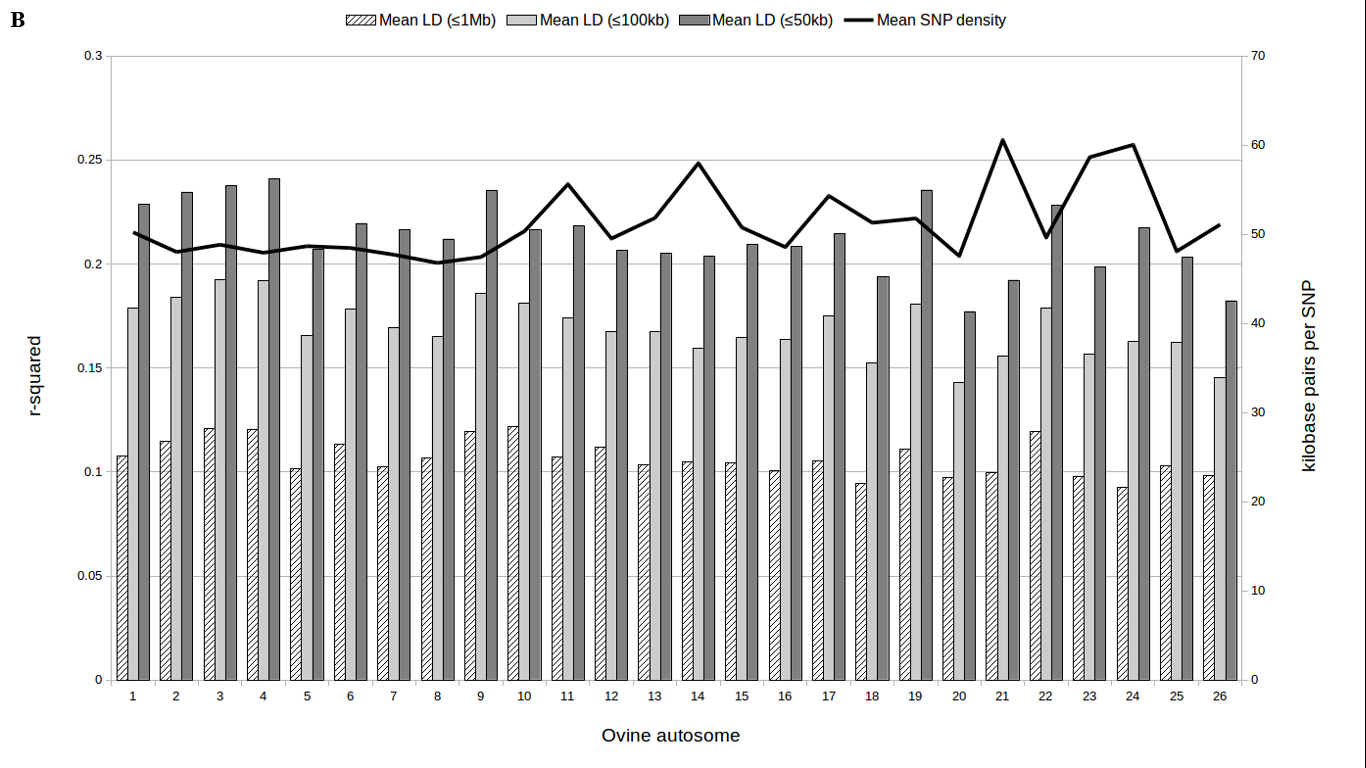

Supplement: Supplementary file 2 [file Image2.TIF]

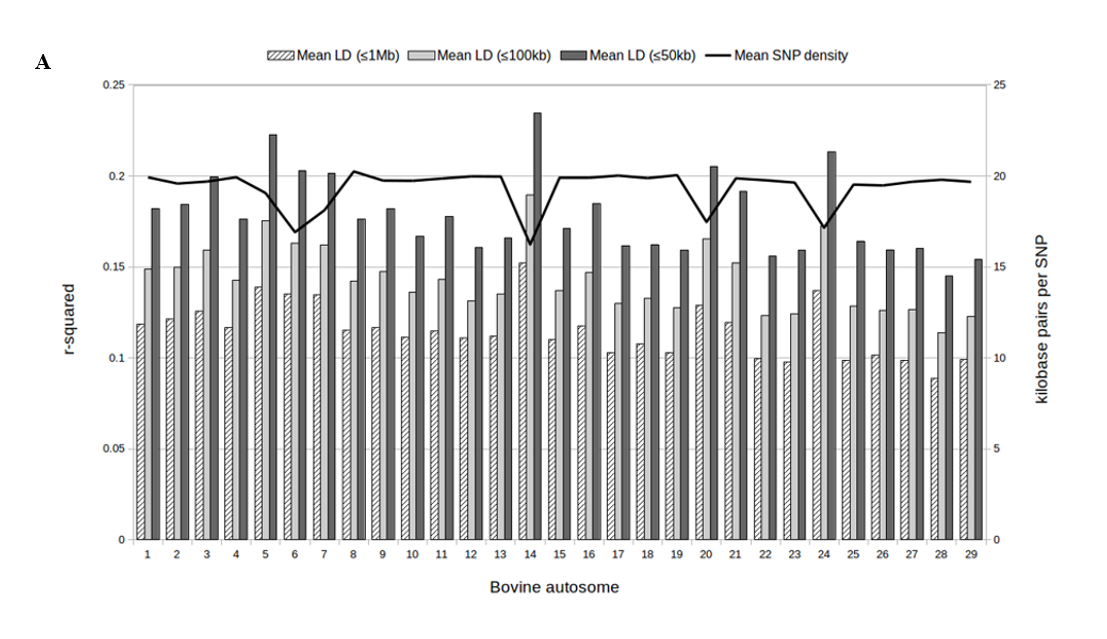

Supplement: Supplementary file 3 [file Image1.TIF]
